# Time, temperature and media: the three keys to improve the recovery of *Campylobacter fetus* subsp. *venerealis* from preputial bull samples

**DOI:** 10.1007/s11259-024-10362-8

**Published:** 2024-04-10

**Authors:** Coral Polo, Teresa García-Seco, Nerea García, Víctor Fernández, Víctor Briones, Alberto Díez-Guerrier, Julio Álvarez, Lucas Domínguez, Marta Pérez-Sancho

**Affiliations:** 1 VISAVET Health Surveillance Centre, Complutense University of Madrid, Av. Puerta de Hierro s/n, 28040 Madrid, Spain; 2 MAEVA SERVET S.L., C/ de la Fragua 3, 28749 Madrid, Spain; 3 Department of Animal Health, Faculty of Veterinary, Complutense University of Madrid, Av. Puerta de Hierro s/n, 28040 Madrid, Spain; 4 Zootecnia Análisis Clínicos Veterinarios S.L.P., C/ Tierra de Campos 24-26, 37008 Salamanca, Spain

**Keywords:** *Campylobacter fetus* subsp. *venerealis*, Isolation, Culture, Transport medium, Enrichment medium, Preputial bull samples

## Abstract

The isolation of *Campylobacter fetus* subsp. *venerealis* (Cfv) from clinical samples is the gold standard for confirming cases of bovine genital campylobacteriosis, an important cause of infertility in cattle and a potential public health concern. Furthermore, isolation is also necessary for the development of autologous vaccines, characterization of strains for antimicrobial susceptibility patterns, etc. Nevertheless, the sensitivity of culture methods is usually low, and there is no standardized protocol to maximize the recovery of Cfv from clinical samples. The aim of the current study is to design a protocol for the culture of Cfv from preputial samples by evaluating the combination of different transport, enrichment and culture media considering the impact of certain factors (time between collection and enrichment, temperature, and use of filters). The use of modified Lander’s transport medium and storing the sample for 24 h at 21 ± 2 °C led to the highest recovery of Cfv CFUs. In contrast, the storage of the samples during 24–48 h in PBS and Thomann rarely allowed the recovery of Cfv regardless of the temperature. The enrichment medium yielding the best results was Preston (significantly higher recovery than Brucella medium), while Cfv could not be isolated with Bolton. Regarding our diagnostic assay (using Lander as transport medium and Preston as enrichment medium), the best protocol in terms of maximizing Cfv recovery as well as limiting contaminations is to culture the samples in i) solid media Preston or Skirrow, and ii) using 0.65 μm filters and incubating plates at 37 °C in microaerophilic conditions.

**Supplementary Information:**

The online version contains supplementary material available at 10.1007/s11259-024-10362-8.

## Introduction

Bovine genital campylobacteriosis (BGC) [caused by *Campylobacter fetus* subsp. *venerealis* (Cfv)] is one of the most important causes of infertility in cattle (Cagnoli et al. 2020; WHOA 2021) and translates into economic losses for farmers (WHOA 2021). Moreover, a potential concern in public health due to Cfv has been also suggested (Patrick et al. 2013; Petersen et al. 2007). Cfv is a venereal pathogen that can be transmitted both by natural breeding and artificial insemination (AI) in cattle (Givens 2018). Infections by Cfv are characterized by early embryonic death and abortions in pregnant cows, while infected bulls are asymptomatic and can act as a reservoir of the pathogen (WHOA 2021). The bacteria proliferate in the preputial crypts increasing in number with the age, thus leading to possible persistent infections in older bulls (Cagnoli et al. 2020).

In many bovine extensive farms where the most frequent reproductive method is natural breeding, performing AI is impractical. The detection of Cfv infections in bulls is an efficient strategy to control Cfv transmission on these farms (Truyers et al. 2014). The bacteriological isolation is considered the gold standard technique for confirming BGC cases (WHOA 2021). However, some limitations of Cfv culture (Chaban et al. 2013; Hedderich et al. 2011; McMillen et al. 2006) reduce their diagnostic performance. Moreover, the drawbacks of Cfv isolation from preputial samples may hamper the implementation of control measures, e.g., the manufacture of autologous vaccines as an alternative to the use of antibiotics. The viability of Cfv in preputial samples seems to be limited by different factors. For example, McMillen et al. (2006) showed that the sensitivity of Cfv culture-based methods was 250-fold lower than the detection of Cfv by real-time PCR when applied on spiked bull smegma samples presumably due to the loss of viability of Cfv over time during the storage of samples. The low success of Cfv isolation from preputial samples may be associated with different factors that can be interconnected, some of which are: i) the susceptibility of Cfv to fluctuations in atmospheric and temperature conditions that may occur during the transport of samples (Koya 2016; McMillen et al. 2006), ii) the extended time frequently elapsed between sampling and culture (Chaban et al. 2013; Lander 1990a, b; Monke et al. 2002), iii) the presence of fast-growing commensal and ubiquitous microorganisms that may act as contaminants (e.g., *Proteus* spp. and *Pseudomonas* spp.) (Clark et al. 1972, 1974; Lander 1990b) in preputial samples, iv) the presence of compounds [e.g., polymyxin B (Marcellino et al. 2015)] in some transport and enrichment media used for the *C. fetus* culture that may limit the growth of some of Cfv strains (Ballabene and Terzolo 1992; Hum et al. 1994), and, v) the susceptibility of *C. fetus* to cell wall rupture due to the use of a Digralsky-spatula technique for plating (Hedderich et al. 2011).

There is a high number of protocols for *C. fetus* isolation from clinical samples (Chaban et al. 2013; Hum et al. 1994; Lander 1990a, b; Monke et al. 2002) offering a wide range of results (some of which are even contradictory) (Chaban et al. 2013; Monke et al. 2002). To our knowledge, a standardized and accurate enough protocol for Cfv culture that may facilitate the development of harmonized schemes for the monitoring of BGC in bulls is currently lacking. The aim of the current study is to develop and assess a complete protocol (from sampling to isolation) that allows maximizing the recovery of Cfv from preputial bull samples. Several transport media, enrichment and culture media were evaluated using different combinations of time, temperature, and filters. The diagnostic performance of the selected protocol with the best results was evaluated using spiked preputial bull samples to assess the impact of the presence of contaminants and the rate of Cfv recovery from field samples.

## Material and methods

### Bibliographic search and methodological considerations

The experiments were designed based on the data collected through a review of the scientific literature on strategies for *C. fetus* isolation from biological samples. The literature selection was based on the follow criteria: i) the abstract of the article or chapter’s book included the use of transport, enrichment and/or culture media for the isolation of Cfv from field samples (prioritizing publications that included a comparison of different media and culture techniques) and ii) protocols were used to recover Cfv from the reproductive tract of cattle. We excluded works published before 1973 (Veron and Chatelain 1973) due to the possible misidentification of *C. fetus* subsp. *venerealis*.

According to the literature: i) the transport media is used to preserve the viability of Cfv from sampling to processing at the laboratory (Chaban et al. 2013; Lander 1990a; Monke et al. 2002), ii) enrichment media is employed to improve the growth of Cfv in presence of other potential microorganisms on the samples (Lander 1990a), and iii) culture plate agar media is used to recover and isolate Cfv colony-forming units (CFU) (Monke et al. 2002). Besides, additional methodologies to decrease the number of non-*Campylobacter* microorganisms isolated have been also reported in the literature, and were considered in this study: i) the use of passive filtration prior to the inoculation of plate agar media (Chaban et al. 2013), and ii) the incubation of plates at 42 °C instead of 37 °C (WHOA 2021). Also, according to previous reports and in order to avoid the rupture of Cfv cells, the use of Digralsky loop technique during culture in plates was limited to 5 seconds (Hedderich et al. 2011).

### From sampling to laboratory (experiment 1): Evaluation of transport media, temperature and time of conservation for *C. fetus*

A total of four transport media were compared (Table 1): i) Weybridge (De Lisled et al. 1982), where the 5-fluorouracil and cycloheximide were replaced by amphotericin B as previously suggested (Koya 2016; Martin et al. 2002; Murinda et al. 2006), ii) Lander (Lander 1990b), where the 5- fluoruracil was replaced by amphotericin B as previously suggested (Koya 2016; Martin et al. 2002; Murinda et al. 2006), iii) Thomann (Harwood et al. 2009), and iv) commercial Stuart transport medium modified (Thermo Fisher Scientific, UK). Phosphate buffered saline (PBS) was used as a control media to assess the viability of Cfv without other compounds as antibiotics and nutrients.

**Table 1:** Composition of each transport media in a final volume of 250 mL

Compound	We	La	Th	St	P	Reference (commercial brand)
Activated carbon powder (g)	1.25	1.25				05105A (Sigma)
Veal infusion broth (g)	6.25					11748812 (Fisher)
Laked blood horse (mL)	17.5	17.5				SR 0048 (Oxoid)
Campylobacter selective supplement	1 vial	1 vial	${~}^{3}\!\left/ \!{~}_{8}\right.$ vial*			SR 0069 (Oxoid)
Amphotericin B [250 μg/mL]** (mL)	10	10	10			A-2942 (Sigma)
Mueller-Hinton broth (g)		5.25				0757-17-6 (DIFCO)
Campylobacter growth suplement		½ vial	½ vial			SR 00232 (Oxoid)
NaCl (g)					2	131659 (Panreac)
KCl (g)					0.05	131494 (Panreac)
Na_2_HPO_4_ (g)					0.36	131679 (Panreac)
K_2_HPO_4_ (g)					0.061	131509 (Panreac)
Stuart (modified) (g)				4		CM0111 (Oxoid)
Nutrient broth N°2 (g)			6.25			CM 0067 (Oxoid)

All compounds were dissolved in distilled water. Is indicated from left to right: Weybridge (We), Lander (La), Thomann (Th), Stuart (St) and PBS (P) pH adjusted to 7.4

*Using 3/8 of a vial is impractical, therefore we recommend producing at least 333.3 mL of media in order to use ½ vial

**In Weybridge medium, the 5- fluorouracil and cycloheximide were replaced by amphotericin B, while in Lander medium the 5- fluoruracil was replaced by amphotericin B. Those modifications were based on Martin et al. (2002), Murinda et al. (2006) and Koya (2016) (Koya 2016; Martin et al. 2002; Murinda et al. 2006) works, due to 5- fluorouracil and cycloheximide seems to be toxic for *C. fetus*

**Fig. 1:**
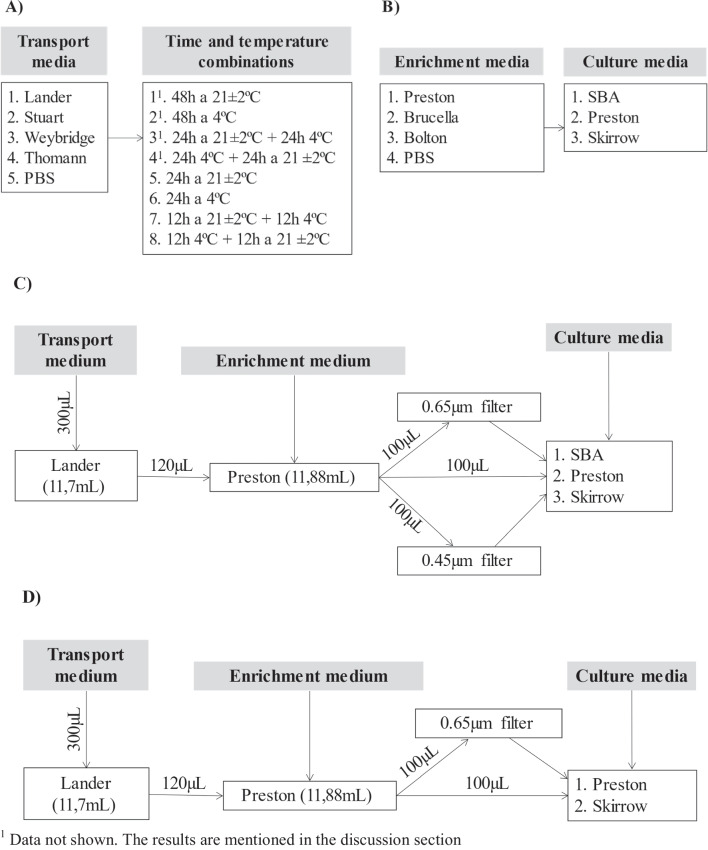
Scheme of the experimental assays **A**) Scheme of the experiment carried out for the selection of transport media (final volume of medium = 12 mL, experimentally inoculated with Cfv, final concentration in each transport media: 3.10^3^ CFU/mL approximately). **B** Scheme of the experiment carried out for the selection of enrichment (cultured at 37 °C during 48 h) and culture media (cultured during 4 days at 37 °C in microaerophilic conditions), previously inoculated with 300 μL of a Lander transport medium with a concentration of 3.10^3^ CFU/mL of Cfv. **C** Workflow of the performance of the experiment 3 with the Group 1 of spiked samples. The Group 1 of spiked samples (*n* = 12) was used to assess the recovery ratio of Cfv under the different conditions of culture according to the workflow showed. The transport medium was not stored during 24 h. **D** Workflow of the performance of the experiment 3 with the Group 2 of spiked samples. The conditions which allowed the highest percentage of Cfv recovery and the lowest percentage of presence of non-Cfv microorganism (based on the results of a logistic regression model, see material and methods section), were tested in the Group 2 of spiked samples (*n* = 20) to assess the differences between selective culture media Preston and Skirrow according to the workflow showed. The group 2 of samples were storage in Lander’s transport medium for 24 h at 21 ± 2 °C

Finally, four combinations of temperature and times for a period of 24 h were evaluated for each transport media as follows (Fig. 1A): i) 48 h at room temperature (RT, 21 °C ± 2 °C), ii) 48 h at refrigeration (RF, 4 °C), iii) 24 h at RT followed by 24 h at RF, and iv) 24 h at RF followed by 24 h at RT. In addition, the same combination of temperatures was tested during 24 h (see Fig. 1A) instead 48 h.

For this study, a suspension of Cfv in 4 mL of PBS was prepared with an approximated concentration of 10^5^ CFU/mL (Supplementary Table 1), a Cfv concentration commonly found in preputial bull samples (Indjein 2013). Then, 300 μL of the suspension were inoculated in 9.7 mL of each transport media to achieve a final concentration of approximately 3 × 10^3^ CFU/mL (except in the case of Stuart medium, see below).

The Cfv strain used in the study (strain ICM18/00036) was provided by Zootecnia S.L. laboratories. The origin of the isolate was from a preputial wash bull sample being identified by MALDI-TOF mass spectrometry as *C. fetus* and by PCR as Cfv according to the PCR protocols published in a previous work (Polo et al. 2021). After each incubation protocol (Fig. 1A), 100 μL of each transport media were inoculated on Skirrow plates (Monke et al. 2002; Skirrow 1977) (Table 2) in duplicates (except in the case of Stuart medium). The Skirrow plates were incubated in boxes of 2.5 L (Thermofisher, UK) at 37 °C during 4 days in microaerophilic atmosphere (8–10% carbon dioxide and 5–10% oxygen) with the use of CampyGen 2.5 L sachets (Thermo Fisher, UK).

**Table 2:** Composition of each enrichment and culture media in a final volume of 250 mL

Compound	Pr	Br*	Bo	Sk	Pr-ag	Bl	Reference (commercial brand)
Nutrient broth NO.2 (g)	6.25					**	CM0067 (Oxoid)
Blood agar base (g)				9.75		**	CM0331B (Oxoid)
Campylobacter agar base (g)					9.25	**	CM0689 (Oxoid)
Dehidrated Brucella broth (g)		7				**	R452662 (Remel)
Bacteriologic agar (g)		0.4				**	A01718 (Condalab)
Bolton broth (g)			6.9			**	CM0983 (Oxoid)
Laked blood horse (mL)	12.5		12.5	12.5	12.5	**	SR 0048 (Oxoid)
Campylobacter selective supplement				½ vial		**	SR 0069 (Oxoid)
Preston campylobacter selective supplement	½ vial				½ vial	**	SR 0204 (Oxoid)
Campylobacter growth supplement	½ vial				½ vial	**	SR 00232 (Oxoid)
Bolton broth selective supplement			½ vial			**	SR0183 (Oxoid)
Bacitracin (g)		0.05				**	11702-5 g (Sigma)
Novobiocin (g)		0.0013				**	N1628 (Sigma)
Amphotericin B (mL)		10				**	A-2942 (Sigma)

All compounds were dissolved in distilled water. The enrichment media were dispensed in tubes (12 mL) while the culture mediums were plating. Is indicated from left to right: Preston enrichment medium (Pr), Brucella enrichment medium (Br), Bolton enrichment medium (Bo), Skirrow plate culture medium (Sk), Preston plate culture medium (Pr-ag) and Blood Sheep agar culture medium (Bl)

* In the Brucella enrichment medium (Br), cycloheximide was replaced by amphotericin B based on Martin et al. (2002), Murinda et al. (2006) and Koya (2016) (Koya 2016; Martin et al. 2002; Murinda et al. 2006) works, due to cycloheximide seems to be toxic for *C. fetus*. Polymyxin B was also removed due to seems to inhibit the growth of *C. fetus* subsp. *fetus* (Public Health Laboratory Network, 2000) and some strains of *C. fetus* subsp. *venerealis* (Ballabene and Terzolo 1992)

**Commercial medium (Biomérieux, 2022. Ref 43,041)

In the case of Stuart medium, which is semisolid, both the transport media and Skirrow agar plates were inoculated using a cotton swab. It was observed in a previous in-house trial that the cotton swab could contain approximately 100 μL. The trial consisted in placing one cotton swab in PBS and then measuring the volume of PBS recovered after centrifuging the cotton end of the swab at 1200 g. Therefore, a cotton swab was immersed in the bacterial suspension for 2 minutes and then the swab was introduced into Stuart tubes. After 24 h, the swab was dragged on a Skirrow plate (this is the only one case in which the culture in plates was not duplicated).

This experiment was performed eight times over three days so that 16 readings were available for each TEM (transport/enrichmentmedium) and temperature-time combination (except for those involving the Stuart medium where culture in plates was not duplicated). The CFU (Colony Forming Units) counts obtained in the Skirrow plates for each transport media and temperature-time combinations were compared through a Poisson model including the transport media (Weybridge, Lander, Thomann, Stuart and PBS as the reference) and the temperature-time combination (24 h RT, 24 h RF, 12 h RT + 12 h RF and 12 h RF + 12 h RT; 48 hour periods were not considered since very limited growth was obtained, see results) as the explanatory variables.

### Processing at laboratory (experiment 2): Evaluation of enrichment and culture media for *C. fetus*

Three enrichment media were tested (Table 2, Fig. 1B): i) Preston (Bolton and Robertson 1982), ii) Bolton (Hunt et al. 2021), and iii) Brucella (Marcellino et al. 2015), where cycloheximide was replaced by amphotericin B as previously suggested (Koya 2016; Martin et al. 2002; Murinda et al. 2006) and Polymyxin B was removed due to its potential inhibition of the growth of some strains of Cfv (Ballabene and Terzolo 1992). PBS was used as negative control to assess the growth of Cfv in a saline medium.

The transport medium selected based on the results of the previous analysis (see results) was inoculated with the same Cfv suspension used in experiment 1 (Supplementary Table 1) in order to achieve a final concentration in transport medium of approximately 3 × 10^3^ CFU/mL in a total volume of 10 mL. Then, 120 μL from this spiked transport medium were inoculated in each enrichment media (final volume = 12 mL, in tubes of 12.5 mL to ensure microaerophilic conditions) and incubated at 37 °C during 48 h (Fig. 1B). After incubation, 100 μL of each enrichment media were cultured in duplicate in three different culture media (Table 2, Fig. 1B): i) Skirrow (Chaban et al. 2013; Monke et al. 2002; Skirrow 1977), ii) Preston agar (Bolton and Robertson 1982) and iii) commercial sheep blood agar (SBA) (Biomérieux, FR). The incubation was carried out at 37 °C during 4 days in microaerophilic atmosphere as above mentioned. The experiment was repeated in three different days yielding six observations for each enrichment-culture media combination. Results per each combination were compared using a Poisson model as explained before with the enrichment-culture media as covariates in order to identify the combination yielding the higher *C. fetus* counts for its consideration in the following experiment (experiment 3) to assess the diagnostic performance of the selected protocol (transport + enrichment media) using spiked bull preputial samples as indicated below.

### Diagnostic performance of selected protocol (experiment 3): Spiked bull preputial samples assessing the impact of temperature of incubation and passive filtration

A total of 32 samples consisting of preputial washings in PBS from 32 breeding bulls raised under extensive management conditions were collected from different Spanish farms. Samples were confirmed as negative to *C. fetus* using a PCR designed by Genetic PCR Solutions (Orihuela, Spain). A total of 4.5 mL of each preputial washings were spiked with 500 µL of a solution containing approximately 10^5^ CFU/mL of the same Cfv strain used in previous experiments (Supplementary Table 1). A first group of samples (Group 1, *n* = 12) was used to assess the recovery rate of Cfv in the presence of possible non-Cfv microorganisms (contaminants) in the sample considering different processing conditions (according to the workflow showed in Fig. 1C): i) apply or not a passive filtration step previous to the inoculation of the culture media [no filters vs. filters with pore size of 0.65 μm or 0.45 μm according to Chaban et al. (2013) (Chaban et al. 2013)], and ii) use of solid culture media [non selective (SBA) or selective (Skirrow 1977)] (Table 2). The culture in solid media was performed at 37 °C vs 42 °C [most Cfv strains are able to grow at 42 °C (WHOA 2021)]. The best protocol based on the combination of temperature, filter usage and culture media was selected based on a logistic regression model that included these three variables as covariates and the isolation of Cfv (yes/no) as the outcome (Supplementary Table 2). In case growth of non-specific bacteria (other than Cfv) was observed in the whole surface of the culture plates, the plate was classified as negative, while in case Cfv was observed in the presence of other non-specific colonies the plate was considered positive.

Based on the results from the first group of samples, a second group (Group 2, *n* = 20) was analysed to further discriminate between the performance of the following processing conditions for recovering Cfv from field samples after the storage of inoculated TEM for 24 h at 21 ± 2 °C according to the workflow showed in Fig. 1D: i) use or not of passive filtration (using filters with a pore size of 0.65 μm), and ii) two selective culture media (Preston vs. Skirrow). The culture in solid media was performed at 37 °C vs. 42 °C.

## Results

### Evaluation of Cfv recovery with the use of transport, enrichment and culture media

**Fig. 2:**
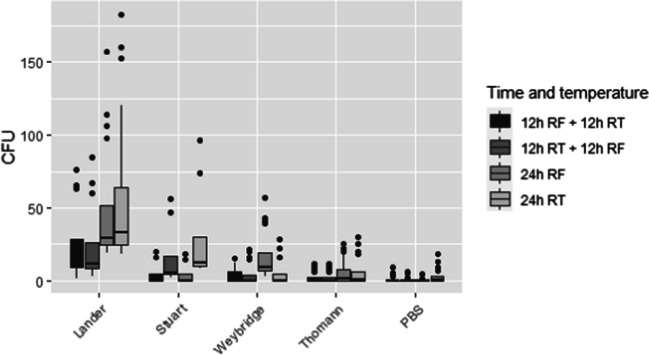
Comparative study of TEM media (experiment 1): CFUs of plate count after the storage of Cfv in different transport medium prior to its culture in Skirrow medium. Boxes indicate the 50% central part of the distribution, whiskers include observations up to 1.5 the interquartile range, and black dots represent outliers. Horizontal bar inside the box indicates the median. The Y axis indicates the number of CFU (Colony-forming unit). The X axis indicates the TEM (Lander, Stuart, Weybridge, Thomann and PBS). Legend indicates time and temperature conditions considered: refrigeration during 12 h (4 °C) follow by 12 h at room temperature (21 °C ± 2 °C) (12 h RF +12 h RT), 12 h at room temperature followed by refrigeration during 12 h (12 h RT + 12 h RF), refrigeration during 24 h at 4 °C (24 h RF) and 24 h at room temperature (24 h RT)

The results of each transport media evaluated are shown in Fig. 2. The viability of Cfv was very low when transport media were stored during 48 h and therefore these results were not considered further.

Samples inoculated in Lander and kept at 24 h at RT yielded the highest CFU counts (median CFU count = 33, interquartile range (IQR) =24.5–63.75), followed by Lander kept at 24 h at RF (median CFU = 29, IQR =24.75–51.5). The highest CFU counts in Stuart were also obtained after 24 h at RT (median CFU = 12, IQR =9.75–29.75), while for Weybridge it was after 24 h at RF (median CFU = 9, IQR = 6.75–18.75). Of note, all Lander plates yielded at least one CFU regardless of the temperature-time considered, while this was also observed only for Stuart after 24 h RT and 12 h RT + 12 h RF. CFU counts in Thomann plates were below 30 regardless of the simulated transport-conservation conditions (Fig. 2). As expected, both transport media and temperature-time conditions were significantly associated with the CFU counts according to the Poisson model, with the Lander and 24 h RT conditions offering the highest predicted CFU counts (Supplementary Table 2).

**Fig. 3:**
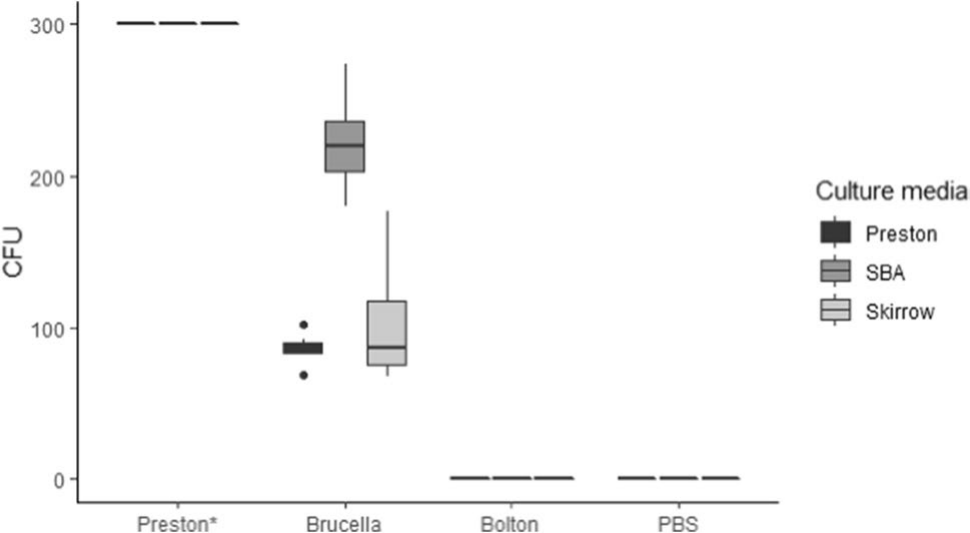
Results of plate count after the incubation at 37 °C during 48 h of different enrichment media previously inoculated with Cfv (experiment 2). Boxes indicate the 50% central part of the distribution, whiskers include observations up to 1.5 the interquartile range, and black dots represent outliers. Horizontal bar inside the box indicates the median. The Y axis indicates the number of CFU (Colony-forming unit). The X axis indicates the enrichment media (Preston, Brucella, Bolton and PBS as negative control). The legend indicates the culture solid media for the colony count (Prestosn, SBA and Skirrow). * The number of CFU was above than 300 in all scenarios; due to the high number of colonies observed, it was not possible to make an exact count

The results of enrichment and culture media are shown in Fig. 3. Preston enrichment medium showed the best results, with all plates yielding >300 CFUs regardless of the culture media (Skirrow, Preston and SBA) used. In contrast, no Cfv colonies were retrieved using Bolton (and PBS) as enrichment medium irrespective of the culture media. When Brucella enrichment medium was used, significantly higher CFU counts were obtained in SBA (median = 220, IQR =203.25–236.75) compared with Skirrow (median CFU = 86.5, IQR =75.25–118, OR from the Poisson model =0.46, 95% CI = 0.42–0.51) and Preston (median CFU = 84.5, IQR =83–90.5, OR = 0.39, 95% CI = 0.35–0.43).

### Diagnostic performance of the selected TEM and enrichment media with spiked bull preputial samples

**Fig. 4:**
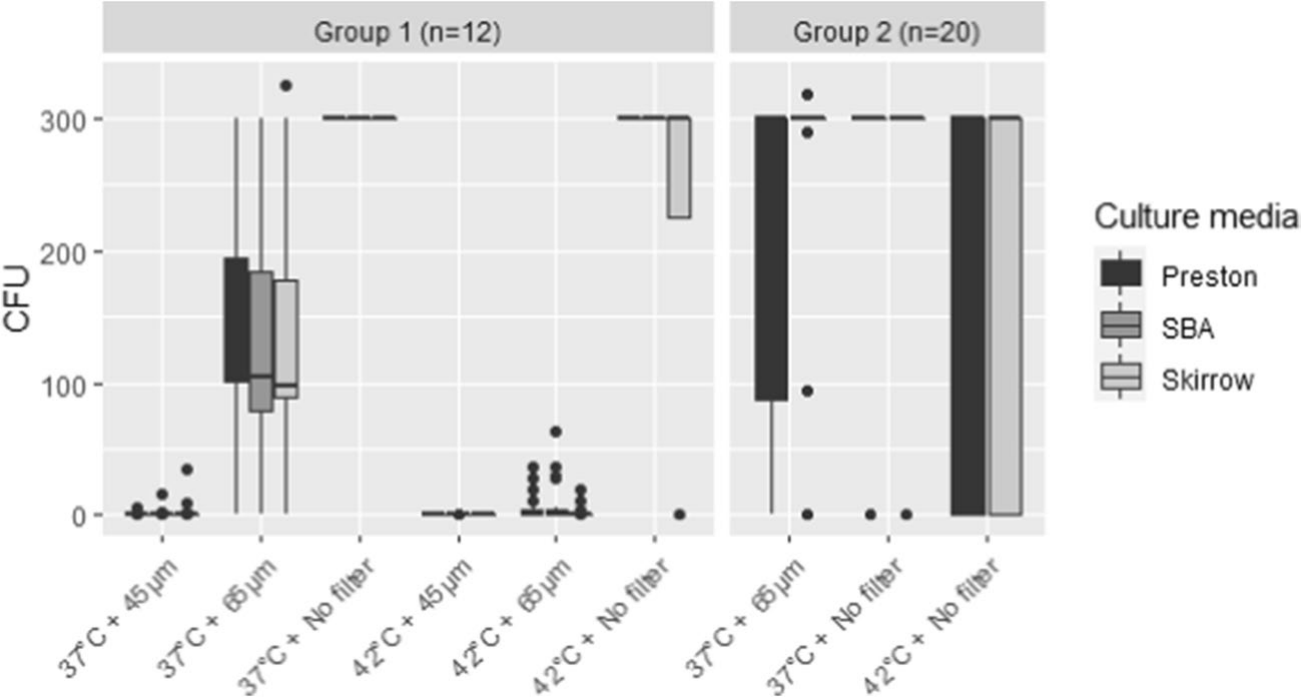
Results of the plate count in the diagnostic assay performed with spiked preputial bull samples with Cfv using Lander medium as TEM and Preston as enrichment medium (experiment 3). Boxes indicate the 50% central part of the distribution, whiskers include observations up to 1.5 the interquartile range, and black dots represent outliers. Horizontal bar inside the box indicates the median. The Y axis indicates the number of CFU (Colony-forming unit). The X axis indicates culture conditions: culture at 37 °C or 42 °C with a previous filtration with a filter of 0.45 μm or 0.65 μm pore diameter or without a previous filtration of the sample (no filter). Those plates with >300 CFU have been considered 300 CFU in the graphic representation. Legend indicates the culture media used for colony count (Preston, SBA and Skirrow). Four plates with undetermined number of Cfv colonies were not included in the Fig.: i) two plates cultured at 37 °C with a previous filtration of the samples with a filter of 0.65 μm in SBA, ii) one plate cultured at 37 °C with a previous filtration of the samples with a filter of 0.65 μm in Skirrow, and iii) one plate cultured at 42 °C with a previous filtration of the samples with a filter of 0.65 μm in SBA

According to our previous results, the media selected for the diagnostic assay were Lander as transport medium and Preston as enrichment medium. However, since the results in the Experiment 2 based on the use of Preston as enrichment medium did not allow to differentiate between the expected performance of the three culture media evaluated, all three options considered (SBA, Preston and Skirrow) were used in the group 1 of samples from Experiment 3. Those plates were inoculated in parallel with and without the passive filtration step (filters of 0.65 μm or 0.45 μm pore size). All plates were incubated in parallel at 37 °C or 42 °C for 4 days. The results are summarized in Fig. 4 and Table 3. The best results in terms of recovery of Cfv were obtained when no filters were used and plates were incubated at 37 °C, while significantly (*p* < 0.001) lower odds of obtaining a positive result were found when plates were incubated at 42 °C and filters were used [although significantly (*p* < 0.001) better results were obtained using 0.65 μm pore size vs. 0.45 μm pore size filters]. In contrast, no significant association between the culture media and the result was found when the effect of the use of filters and the temperature was considered in the model. Regarding the growth of non-Cfv bacteria (contaminants), use of SBA and not using filters resulted in a higher proportion of contaminated plates compared with Skirrow and Preston and using 0.65 and especially 0.45 μm filters, while a more limited impact of the temperature was observed (Table 3).

**Table 3:** Results of the diagnostic assay performed with spiked preputial bull samples with *C. fetus* subsp. *venerealis* (strain ICM18/00036) using Lander medium as TEM and Preston as enrichment medium (experiment 3)

				No filter	0.65 μm	0.45 μm
Culture media. Group 1 (*n* = 12)	Preston	% of CFU recovery	**37 °C**	100	100	20.8
			**42 °C**	100	29.2	0
		% of contaminants	**37 °C**	12.5	12.5	4.2
			**42 °C**	4.2	0	0
	SBA	% of CFU recovery	**37 °C**	100	87.5	16.6
			**42 °C**	100	45.8	8.3
		% of contaminants	**37 °C**	58.3	37.5	0
			**42 °C**	54.2	25	0
	Skirrow	% of CFU recovery	**37 °C**	100	95.8	16.6
			**42 °C**	75	25	0
		% of contaminants	**37 °C**	16.6	8.3	0
			**42 °C**	8.3	8.3	0
				No filter	0.65 μm	
Culture media. Group 2 (*n* = 20)	Preston	% of CFU recovery	**37 °C**	85	90	
			**42 °C**	72.5	NA	
		% of contaminants	**37 °C**	35	12.5	
			**42 °C**	30	NA	
	Skirrow	% of CFU recovery	**37 °C**	85	82.5	
			**42 °C**	70	NA	
		% of contaminants	**37 °C**	42.5	17.5	
			**42 °C**	30	NA	

It is shown the percentage of *C. fetus* subsp. *venerealis* recovery (% of CFU recovery, that corresponds to the % of plates positive for Cfv in relation to the total plates) and the percentage of contaminated plates (% of contaminants, that correspond to the % of plates positive for Cfv related to the total plates) of the group 1 of samples (*n* = 12) and group 2 (*n* = 20) in Preston, sheep blood agar (SBA) and Skirrow media under different conditions: culture at 37 °C or 42 °C without a previous passive filtration of the sample (no filter) or with a previous passive filtration with a filter of 0.65 μm or 0.45 μm pore size. NA: conditions not analysed. All combinations of evaluated conditions were cultured by duplicate for each sample

The results from the analysis of samples in Group 2 of experiment 3, processed at 37 or 42 °C with or without passive filtration with 0.65 μm pore size filters using selective media (Preston and Skirrow), are summarized in Fig. 4 and Table 3. According to group 1 samples results, 0.45 μm pore size filters were not included due to their impact in the Cfv recovery. A significantly (*p* < 0.001) higher probability of recovering Cfv was found when plates were cultured at 37 °C (OR = 2.29, 95% CI 1.06–5.14) while the use or not of 0.65 μm pore size filters and the specific selective media used did not have a significant (*p* > 0.5) impact on the Cfv recovering result. In contrast, the use of 0.65 μm filters resulted in a lower proportion of contaminated plates, while the media and the temperature had a minor impact on the detection of non-Cfv growth.

## Discussion

The isolation of Cfv is still considered the gold standard for confirmation of BGC, an important cause of infertility in cattle that translates onto economic losses for farmers (Cagnoli et al. 2020; WHOA 2021) and a potential concern for public health (Wagenaar et al. 2014). The isolation of Cfv from clinical samples is necessary to develop autologous vaccines as an alternative to the use of antibiotics, and to characterize strains to identify virulence factors, etc. Nevertheless, the environmental viability of Cfv is limited (Chaban et al. 2013; Koya 2016; Monke et al. 2002), this can impact the probability of isolating the pathogen from clinical samples reducing the diagnostic efficiency of BGC through culture. Different strategies have been developed to overcome these drawbacks (Chaban et al. 2013; Hum et al. 1994; Lander 1990a, b; Monke et al. 2002). However, to our knowledge, a standardized protocol to maximize the recovery of Cfv from preputial bull samples has not been established, thus limiting the reliability of BGC culture-based diagnosis.

We compared different TEM, enrichment, and culture media, as well as different protocols for sample processing and culture: i) different times and temperatures for inoculated TEM to assess the best conditions to maximize the viability of Cfv, and ii) filters and different temperatures of incubation to assess the best strategy to reduce the presence of non-*C. fetus* microorganism. The aim of this study was to establish a complete protocol that maximizes the recovery of Cfv isolates from preputial bull samples to improve the sensitivity of culture. Cfv is a fastidious bacteria (WHOA 2021); for this reason, we limited the use of Digralsky loop to 5 (Hedderich et al. 2011). At this point, it is important to indicate that the bacterial count was relatively variable between duplicates as previously reported (Koya 2016).

According to our results, the modified Lander medium (Table 1) showed the best recovery result of Cfv (Fig. 1A), although differences on methodological approaches due to different consistency media (liquid versus semisolid) should be considered. Modified Lander medium lacked 5-fluoracil in its composition (Lander 1990b) since it may limit the growth of *C. fetus* as previously suggested (Ballabene and Terzolo 1992; Martin et al. 2002; Murinda et al. 2006). However, this modified Lander medium contained amphotericin B (Table 1) as described in previous works (Koya 2016; Martin et al. 2002; Murinda et al. 2006). Changes of temperature reduced the recovery of Cfv (Fig. 1A), as previously reported by Koya (2016) (Koya 2016). Thus, increasing efforts in keeping a constant temperature during transport and preservation of samples up to 24 h using Lander as transport medium, could significantly improve the viability of Cfv. According to our results, sample conservation at room temperature during 24 h, allows to recover higher Cfv CFUs than under refrigeration. This is an important point to consider by clinical veterinarians as well as in laboratory protocols, since the use of refrigeration of samples to improve the viability of microorganisms is a common procedure. The virtual absence of recovery of Cfv after 48 hours is consistent with the previously cited poor survival of this pathogen (Chaban et al. 2013; Monke et al. 2002) and highlights the importance of processing samples in the laboratory as soon as possible after collection to avoid false negative results (Monke et al. 2002). Finally, PBS is a common media for preputial bull sampling (WHOA 2021) commonly used for transportation of samples. However, according to our results, the storage of preputial samples in PBS during 24 h rarely allowed the recovery of Cfv colonies (CFU mean < 1) (Fig. 2), a similar result to that observed in previous published studies with other saline media (Monke et al. 2002).

Regarding the evaluation of enrichment media (experiment 2), Preston medium showed the best results, and the recovery of Cfv was possible in the 100% of cases, yielding the highest CFU counts (>300 CFUs) compared to the other media regardless of the culture media used after enrichment (Fig. 3). Our modified Brucella enrichment medium could be considered a suitable alternative since the mean number of CFU observed was high (Fig. 3). In this medium (Marcellino et al. 2015), cycloheximide was replaced by amphotericin B due to its potential toxic effect for *C. fetus* (Koya 2016; Martin et al. 2002; Murinda et al. 2006), and polymyxin B was removed due to its potential to inhibit the growth of some Cfv strains (Ballabene and Terzolo 1992). Finally, no Cfv colonies were recovered when Bolton or PBS were used as enrichment. Although the Bolton enrichment medium is commonly used for the isolation of other *Campylobacter* species from food and water (Hunt et al. 2021), according to our results, it is not suitable for Cfv isolation from bull preputial samples (Fig. 3).

In order to determine the most suitable culture conditions for clinical samples to maximize Cfv recovery and to limit the growth of non-*C. fetus* microorganisms that may reduce the sensitivity of Cfv isolation, two groups of Cfv spiked preputial samples (Group 1 *n* = 12, and Group 2 *n* = 20) were cultured under different protocols considering three variables combined: culture temperature, use or not of filters and different solid culture media. No significant reduction in the presence of contaminants was observed within both groups of samples regardless the temperature of culture considered, whereas incubation at 42 °C reduced the recovery of Cfv, an effect also observed when passive filtration was applied (Table 3, Fig. 4). Thus, according to our results, incubation at 42 °C is not recommended as an alternative to 37 °C, since it had a limited impact in reducing the growth of contaminants but significantly decreased the probability of Cfv isolation, especially in combination with the use of filters. It has been reported that the use of filters reduce the bacterial recovery (Clark et al. 1974). Furthermore, considering that not all Cfv strains are able to grow at 42 °C, culture at this temperature may contribute to false-negative results even in the presence of a low presence of non-*C. fetus* microorganisms.

The use of 0.45 μm and 0.65 μm filters is a strategy reported to reduce the presence of contaminants in Cfv and other *Campylobacter* species isolation protocols (Chaban et al. 2013; WHOA 2017). In agreement with these findings, our results demonstrated that the presence of contaminants significantly decreased with the use of 0.45 μm filters. However, this approach also decreased the probability of Cfv isolation (Table 3), especially when a non-selective culture medium (SBA) was used, so the use of 0.45 μm filters is discouraged. On the other hand, the use of 0.65 μm filters, according to the results of the groups of samples 1 and 2, does not significantly affect the isolation capacity of Cfv but can help to reduce the presence of contaminants both on selective media as well as non-selective media (SBA) (Table 3).

Regarding the results obtained when culture was performed at 37 °C, Group 1 of samples showed that SBA (medium without antibiotics) has a Cfv recovery capacity (100% without the use of filters and 87.5% with the use of 0.65 μm filters) similar to the selective media evaluated Preston (100% without the use of filters and 100% with the use of 0.65 μm filters) and Skirrow (100% without the use of filters and 95.8% with the use of 0.65 μm filters). However, a higher percentage of contaminations was observed in SBA (Table 3), therefore its use is discouraged. On the other side, no statistically significant differences were observed in the results of sample groups 1 and 2 between both selective media in terms of Cfv isolation rate (Table 3) (considering the effect of temperature and the use of filters), being both, Preston and Skirrow media, suitable for Cfv isolation from preputial wash samples. Our results do not seem agree with the information showed by Chaban et al. (2013) (Chaban et al. 2013), where the best option for the recovery of Cfv was the use of filters of 0.65 μm pore size in SBA medium compared with the use of Skirrow without filtration. Nevertheless, it is important to note that the work of Chaban et al. (2013) (Chaban et al. 2013) did not include transport or enrichment media.

Although further studies should be performed using different Cfv strains, based on the results obtained in the present study, we recommend the use of Lander stored at constant temperature [preferably at room temperature (21 °C ± 2 °C) rather than under refrigeration (4 °C)] for a transport period of up to 24 h from sampling to culture as the transport medium-temperature best conditions. The recommended protocol then includes the inoculation of Preston enrichment medium incubated for 48 h at 37 °C in microaerophilic conditions. For culture media we recommend the use of Preston or Skirrow agar plates incubated at 37 °C for four days under microaerophilic conditions. While the use of 0.65 μm pore size filters can reduce the growth of non Cfv colonies (contaminations), its possible effect on reducing the sensitivity of the Cfv isolation protocol in some scenarios should be considered.

## Supplementary Information

ESM 1 (DOCX 14 kb)

ESM 2 (DOCX 18 kb)
