# Supplementary material for: Time, temperature and media: the three keys to improve the recovery of Campylobacter fetus subsp. venerealis from preputial bull samples
Source: Vet Res Commun. 2024 Apr 10;48(4):2109–19. doi: 10.1007/s11259-024-10362-8 (PMC11637040; doi:10.1007/s11259-024-10362-8)
Supplement: Supplementary file 1 — (DOCX 14 kb) [file 11259_2024_10362_MOESM1_ESM.docx]

Supplementary Table 1. The concentration of *Campylobacter fetus* was determined using a McFarland densitometer (DEN-1B McFarland densitometer, Biosan) and colony counts were performed on Blood agar plates (Biomérieux, 2022. Ref 43041). A *C. fetus* subsp. *venerealis* suspension of 1 McFarland in PBS was measured by densitometry, and then serial dilutions (1/10) were made. Finally, 100 µL of each dilution was cultured on blood agar plates and incubated for 4 days at 37°C in microaerophilic conditions (8-10% carbon dioxide and 5-10% oxygen). Extrapolating the count results at 1McFarland x 10^-3^ and 10^-4^ was established using the following calculations:

| **Dilution in PBS** | **Colony count** | **CFU/mL** |
| --- | --- | --- |
| 1McFarland | > 1000 | 10^6^ |
| 1McFarland x 10^-1^ | > 1000 | 10^5^ |
| 1McFarland x 10^-2^ | > 1000 | 10^4^ |
| 1McFarland x 10^-3^ | 210 | 10^3^ |
| 1McFarland x 10^-4^ | 62 | 10^2^ |
