# Supplementary material for: Time, temperature and media: the three keys to improve the recovery of Campylobacter fetus subsp. venerealis from preputial bull samples
Source: Vet Res Commun. 2024 Apr 10;48(4):2109–19. doi: 10.1007/s11259-024-10362-8 (PMC11637040; doi:10.1007/s11259-024-10362-8)
Supplement: Supplementary file 2 — (DOCX 18 kb) [file 11259_2024_10362_MOESM2_ESM.docx]

Supplementary Table 2. Coefficients and ORs of the Poisson model used with the information of the experiment 3, group 1 of samples to assess the relation between CFU counts observed in Skirrow media depending on the TEM and temperature-time. 12RF12RT: refrigeration (4 ºC) during 12h followed by 12h at room temperature (21ºC±2ºC); 12RT12RF: 12h at room temperature followed by refrigeration for 12h; 24RF: refrigeration during 24h; and 24RT: 24h at room temperature. Reference category: PBS and 24RT.

| TEM | Temperature-time | Estimate | Std. Error | OR | 95% CI |
| --- | --- | --- | --- | --- | --- |
|  |  | 1.159 | 0.140 |  |  |
| Lander | 12RF12RT | 2.050 | 0.149 | 7.76 | 5.86-10.51 |
| Lander | 12RT12RF | 2.027 | 0.149 | 7.59 | 5.73-10.28 |
| Lander | 24RF | 2.745 | 0.144 | 15.57 | 11.86-20.92 |
| Lander | 24RT | 2.940 | 0.144 | 18.92 | 14.44-25.39 |
| Stuart | 12RF12RT | 0.345 | 0.218 | 1.41 | 0.92-2.16 |
| Stuart | 12RT12RF | 1.629 | 0.165 | 5.10 | 3.71-7.11 |
| Stuart | 24RF | 0.258 | 0.223 | 1.29 | 0.83-1.99 |
| Stuart | 24RT | 2.225 | 0.154 | 9.25 | 6.9-12.66 |
| Weybridge | 12RF12RT | 0.000 | 0.198 | 1.00 | 0.68-1.48 |
| Weybridge | 12RT12RF | 0.331 | 0.184 | 1.39 | 0.97-2 |
| Weybridge | 24RF | 1.674 | 0.153 | 5.33 | 3.99-7.27 |
| Weybridge | 24RT | 0.546 | 0.176 | 1.73 | 1.23-2.45 |
| Thomann | 12RF12RT | -0.376 | 0.219 | 0.69 | 0.44-1.05 |
| Thomann | 12RT12RF | -0.321 | 0.216 | 0.73 | 0.47-1.1 |
| Thomann | 24RF | 0.601 | 0.174 | 1.82 | 1.3-2.58 |
| Thomann | 24RT | 0.653 | 0.173 | 1.92 | 1.38-2.71 |
| PBS | 12RF12RT | -1.099 | 0.280 | 0.33 | 0.19-0.56 |
| PBS | 12RT12RF | -1.224 | 0.294 | 0.29 | 0.16-0.51 |
| PBS | 24RF | -1.986 | 0.403 | 0.14 | 0.06-0.28 |
